# Steatosis in South African women: How much and why?

**DOI:** 10.1371/journal.pone.0191388

**Published:** 2018-01-19

**Authors:** Nitien H. Naran, Mark Haagensen, Nigel J. Crowther

**Affiliations:** 1 Department of Chemical Pathology, National Health Laboratory Service, Faculty of Health Sciences, University of the Witwatersrand, Johannesburg, South Africa; 2 Department of Radiology, Donald Gordon Medical Centre, University of the Witwatersrand, Johannesburg, South Africa; University of Cape Town, SOUTH AFRICA

## Abstract

**Background:**

Globally, steatosis is the commonest type of liver pathology and is closely associated with obesity and the metabolic syndrome. Obesity is common in urban African females but no data is available on hepatic fat content in this population group when compared to other ethnic groups. The aim of this study was therefore to compare hepatic fat content in woman from different ethnic groups in South Africa and to characterise the principle determinants of liver fat.

**Materials and methods:**

A convenience sample of 106 (48 Indian, 29 African and 29 Caucasian) female volunteers aged 20–60 years and having no history of cardiometabolic disorders were recruited. Hepatic fat was determined from CT scans using the liver-spleen attenuation ratio (LAR), which decreases with increasing levels of hepatic fat. Anthropometric and cardiometabolic parameters were measured with insulin resistance determined using the HOMA index and dysglycaemia defined as fasting glucose ≥5.60 mmol/L.

**Results:**

The African subjects had significantly lower hepatic fat content (LAR as median [interquartile range]: 1.35 [1.28, 1.41]) than the Indian (1.22 [1.10, 1.35]; p<0.005) and Caucasian (1.27 [1.16, 1.33]; p<0.05) females even though they had significantly higher BMIs than both groups (p<0.0005 and p<0.05, respectively). Linear regression showed that: subcutaneous abdominal fat was a significant (unstandardised β = 0.007; p = 0.03) negative, whilst insulin resistance (β = -0.97; p = 0.01) and dysglycaemia (β = -3.58; p = 0.01) were significant positive determinants of liver fat; higher hepatic fat levels in subjects with the metabolic syndrome were explained by insulin resistance and dysglycaemia.

**Discussion:**

African ethnicity is associated with low liver fat content. Subcutaneous abdominal fat protects against steatosis, possibly by acting as a triglyceride reservoir. Insulin resistance and dysglycaemia lead to greater hepatic fat deposition and explain higher liver fat levels in subjects with the metabolic syndrome. These observations must be further investigated in longitudinal surveys.

## Background

Recent studies have shown that accumulation of lipids within non-adipose tissue may result in a shift in both glucose and lipid homeostasis [1]. Furthermore, it has been shown that the presence of excessive lipids within the liver is able to decrease the efficiency of insulin signalling resulting in insulin resistance [2]. There are many causes of lipid accumulation in the liver including chronic autoimmune disturbances, Wilson’s disease, alcohol intake, hepatitis C, and certain pharmacological agents, but metabolic disturbances are considered to be the primary cause of fatty liver disease.

The term non-alcoholic fatty liver disease (NAFLD) denotes a range and severity of liver diseases starting from simple fatty liver (steatosis) progressing to non-alcoholic steatohepatitis (NASH) to cirrhosis and ultimately hepatocellular carcinoma, in individuals who do not consume excessive amounts of alcohol. Steatosis involves the accumulation of fat, particularly triglycerides within hepatocytes. Initially, steatosis was thought to be relatively harmless, however it is now known that hepatic fat accumulation can cause an inflammatory response followed by necrosis, fibrosis and cirrhosis, ultimately leading to hepatocellular carcinoma [3]. The aetiology of NASH is complex with many factors, (demographic, behavioural and biological) having been linked with its aetiology [4–7].

Chronic liver diseases have been shown to be a major health risk and there may be substantial disparities in related mortality across ethnicities, genders, socioeconomic standings and geographical regions. Thus, Nguyen et.al. [4] suggested that some ethnic groups may be disproportionately more vulnerable than others in developing NAFLD through socioeconomic and cultural factors, thereby implying that in order to fully understand the aetiology of NAFLD prospective studies should investigate the contribution of ethnicity and socioeconomic status (education, income, and occupational status), before any meaningful conclusions can be drawn.

The development of NAFLD has been reported to be strongly associated with obesity, particularly visceral adiposity [5,6] and type 2 diabetes [7]. These factors are two major contributors to the development of the metabolic syndrome, and since ethnicity, diet and socioeconomic status have been shown to play pivotal roles in the development of the metabolic syndrome, they may also contribute significantly to the susceptibility of NAFLD. While the pathophysiological mechanisms linking NAFLD with obesity and diabetes are not clearly elucidated, it is thought that increased visceral obesity leads to higher levels of fatty acid in the portal vein and hepatocyte fat deposition [8].

Several studies have shown that increased liver enzymes and ultrasonographic appearance of hepatic steatosis were predictors of cardiovascular disease independent of conventional risk factors [9]. Furthermore, it has been shown that subjects with NAFLD have higher carotid intima-media thickness, impaired endothelial function and lower concentrations of adiponectin [10].

A recent national survey in South Africa reported that almost 40% of women were found to be obese compared to 10% in males [11]. A closer look at the prevalence of obesity in South Africa shows that it is highly prevalent among the African population and lowest in the Indian population [11,12]. Studies in South Africa have also shown that the Indian population have a higher waist-to-hip ratio than both Caucasian and black South Africans [13] and a higher level of visceral fat in Indian compared to black African South African females [14]. However, there are no studies as yet on the prevalence and determinants of NAFLD and its association with insulin resistance and the metabolic syndrome in the female South African population. Therefore, the principal aim of this study was to determine the main predictors of hepatic fat levels in a population of African, Indian and Caucasian women. Only women were used in this study due to the high prevalence of female obesity in the South African population [11,12]. The secondary aims of the study were to compare the degree of hepatic fat deposition across these population groups, to observe the association between hepatic fat content and risk markers of cardiometabolic disease and to determine whether metabolic syndrome is associated with higher intrahepatic fat content.

## Materials and methods

### Subjects

A convenience sample of 106 (48 Indian, 29 African and 29 Caucasian) female volunteers were recruited from the staff working at the Charlotte Maxeke Johannesburg Academic Hospital and the Medical School of the University of Witwatersrand. Participants were asked to give details of colleagues who they thought met the inclusion criteria for the study and these were interviewed by the principal investigator (PI). Prospective participants were also asked to tell colleagues about the study and to contact the PI if they wished to be enrolled. Subjects were asked about their current medical history and were excluded from the study if they had been diagnosed by a health care professional with hypertension, diabetes, dyslipidaemia or any form of heart disease, and were receiving medication for these disorders. Only subjects between the ages of 20–60 years were included in the study. The study was explained to the volunteers and written informed consent obtained. The study was approved by the University of the Witwatersrand Human Research Ethics Committee.

### Anthropometric measurements and assessment of hepatic fat

Age, blood pressure, weight, height and waist circumference were recorded. Waist circumference was taken as the midpoint between the lower rib margin and the iliac crest. Visceral and subcutaneous fat depots were measured by computerized tomography (CT). Thus, a multi-detector row CT scan (Brillliance 6, Philips, Netherlands) was obtained from the diaphragm to just below the lumbo-sacral junction, consisting of 5 mm contiguous slices (KVp 90, mAs170-200, FOV 50cm, matrix 512x512). The images were transferred to an Apple work station using the open software program Osirix [15] to process the images. Using multi-planar viewing a volume of interest (VOI) was created from just below the pedicles of L4 to the level of the L4-L5 disc. This was saved separately and utilised for the intra and extra abdominal fat measurements.

Hepatic fat content was measured by selecting a single axial slice through the mid-level of the liver and spleen. Three regions of interest were placed over the liver parenchyma, another 3 over the spleen and one in each of the erector spinae muscles. The Hounsfield units (HU) of each of the regions of interest were recorded as a measurement of tissue density. Liver and spleen attenuation measurements in Hounsfield units were taken at each of the regions of interest and used to calculate the liver-to-spleen attenuation ratio (LAR) [16]. The LAR decreases with increasing hepatic lipid deposition.

### Blood analyte measurements

Fasting blood samples were collected and serum lipogram (total serum cholesterol, triglycerides and HDL) concentration and fasting blood glucose levels were determined on the ADVIA^®^ chemistry system (Siemens, Munich, Germany). Low density lipoprotein (LDL) cholesterol was determined indirectly using the Friedewald formula [17], and fasting insulin levels were measured using the ADVIA^®^ Centaur system. Insulin resistance was quantified using the HOMA method [18]. Dysglycaemia was diagnosed based on a fasting glucose level of ≥5.6 mmol/L whilst metabolic syndrome was diagnosed using the harmonised guidelines [19].

### Assessment of physical activity, alcohol intake and education level

Physical activity was measured using an activity monitor (SenseWear Pro_2_ Armband, BodyMedia, Pittsburg, PA, USA), which was worn for a maximum of 7 days. The monitor gave values for total energy expenditure per day, energy expenditure per day due to physical activity, number of steps walked per day and metabolic-equivalent scores (METs).

Alcohol intake was assessed by asking all participants whether they consumed alcohol at all, occasionally (not regularly, at social events only), or regularly (at least once every 1 or 2 weeks). No participants reported regular alcohol intake with all subjects who did report taking alcohol saying this was occasional. Alcohol intake was therefore coded as a simple ‘yes’ or ‘no’.

Each participant was asked for their highest level of educational attainment and was then coded as receiving or not receiving tertiary education.

### Statistical analyses

Normally distributed data was expressed as mean ± SD whist non-parametric data was expressed as median [interquartile range]. The latter variables were log transformed to normality before use in t-tests or Pearson correlation analyses. The LAR values could only be normalised by squaring them. Variable means were compared across the 3 ethnic groups via ANOVA followed by Tukeys *post hoc* test whilst variable means were compared across 2 groups using Students unpaired t-test. An ANOVA was also used to compare blood pressure, insulin resistance, lipid, glucose and insulin levels across tertiles of hepatic fat. The principle determinants of the levels of hepatic fat were isolated using a multivariable linear regression analysis. Study variables chosen based on scientific plausibility for influencing liver fat levels were correlated with LAR using Pearson correlation. The variables chosen were: ethnicity, age, BMI, waist, visceral and subcutaneous fat, HOMA, dysglycaemia, metabolic syndrome, triglycerides, total energy expenditure per day, energy expenditure per day due to physical activity, number of steps walked per day, metabolic-equivalent scores, education, smoking and alcohol intake. Variables that correlated with LAR at p<0.50 were included in the multivariable regression model. Backward, stepwise regression was then performed to leave only predictor variables that had significant (p<0.05) beta values. A similar process was used to find the principal determinants of HOMA. The scientifically plausible variables that were chosen and analysed via Pearson correlation against HOMA were the same as those used for LAR but excluding metabolic syndrome, dysglycaemia, HOMA, and lipids but including LAR.

A sample size was calculated based on the main statistical method to be used, which was a multivariable linear regression analysis to isolate the main predictors of hepatic fat content. Assuming that there would be a maximum of 7 independent variables in the final regression model, with an effect size of 0.20, a statistical power of 90% and a p-level of 0.05, the estimated sample size was 99. The full dataset for this study is included as supporting information (see S1 Data file Naran et al).

The Statistica software package was used for all statistical analyses (version 12, StatSoft, Tulsa, USA).

## Results

### Anthropometry, biochemical parameters and activity levels in Indian, African and Caucasian subjects

The anthropometric measurements, biochemical parameters and the mean daily energy expenditure of the Indian, African and Caucasian subjects are shown in Table 1. There were no significant differences across the 3 groups for age, systolic and diastolic blood pressure, fasting blood glucose, total cholesterol, triglyceride, low density lipoprotein (LDL) and high density lipoprotein (HDL) concentrations or tertiary education levels. The prevalence of neither dysglycaemia nor smoking differed significantly across the groups, whereas a significantly higher percentage (78.6%) of Caucasian subjects occasionally consumed alcohol than did the African (20.7%; p<0.0005) or the Indian (10.4%; p<0.0005) subjects. The African subjects had a significantly higher BMI and waist circumference as compared to the Indian (p<0.0005 and p<0.005 respectively) and Caucasian (p<0.05 and p<0.0005 respectively) subjects. The CT scans showed that the African subjects had significantly (p<0.05) higher subcutaneous fat depots than the Caucasian subjects, whereas the Indian subjects had higher visceral fat depots than the African and Caucasian subjects but it did not reach significance. The Indian and Caucasian subjects had a significantly (p<0.005 and p<0.05, respectively) lower liver to spleen attenuation ratio (LAR) as compared to the African subjects indicating that the Indian and Caucasian populations had significantly higher hepatic lipid content than the African subjects. The insulin concentrations were significantly (p<0.05 for both) higher in the Indian and Caucasian subjects as compared to the African subjects. Therefore, when using the HOMA index to determine insulin resistance, the Indian and Caucasian subjects were shown to be significantly more insulin resistant than the African subjects.

**Table 1:** Comparison of study variables between population groups.

Variables	Indian	African	Caucasian
***Age (years)***	38.3 ± 10.4	37.3 ± 12.8	35.9 ± 14.4
***BMI***	24.7 [21.5, 27.2]***	30.1 [25.7, 34.3]	26.2 [22.1, 28.3]*
***Waist (cm)***	80.7 ± 11.9**	87.1 ± 14.3	78.0 ± 8.91***
***Visceral fat (cm***^***3***^***)***	82.4 [45.9, 123]	76.9 [35.9, 158]	44.2 [27.9, 108]
***Subcutaneous fat (cm***^***3***^***)***	361 [237, 435]	443 [325, 643]	313 [213, 414]*
***Liver: spleen ratio***	1.22 [1.10, 1.35]**	1.35 [1.28, 1.41]	1.27 [1.16, 1.33]*
***Insulin (pmol/L)***	51.1 [34.2, 87.5]*	28.2 [19.0, 64.5]	52.9 [28.8, 70.7]*
***Glucose (mmol/L)***	4.80 [4.60, 5.00]	4.90 [4.70, 5.27]	4.70 [4.50, 5.10]
***HOMA***	1.63 [0.98, 2.50]*	0.86 [0.60, 2.06]	1.64 [0.95, 2.38]*
***Triglycerides (mmol/L)***	1.02 [0.69, 1.52]	0.80 [0.60, 1.17]	1.01 [0.78, 1.43]
***Total cholesterol (mmol/L)***	4.76 [4.30, 5.34]	4.56 [3.77, 5.00]	4.83 [4.28, 5.77]
***LDL (mmol/L)***	2.67 [2.35, 3.22]	2.55 [2.00. 3.20]	2.80 [2.30, 3.40]
***HDL (mmol/L)***	1.43 [1.22, 1.66]	1.50 [1.18, 1.70]	1.67 [1.40, 1.82]
***Systolic bp (mmHg)***	119 [109, 126]	120 [110, 130]	113 [108, 120]
***Diastolic bp (mmHg)***	80.0 [70.0, 84.0]	76.0 [70.0, 80.0]	70.0 [70.0, 79.0]
***Dysglycaemia (%)***	14.6	13.8	6.90
***Metabolic syndrome (%)***	27.1^++^	17.2	0.00
***Tertiary education (%)***	64.7	60.7	77.8
***Walking (steps per day)***	8927 [6048, 11063]*	10871 [7424, 12820]	8056 [6881, 9846]
***Total energy expenditure (cals/day)***	2198 [1957, 2623]**	2646 [2211, 3134]	2283 [2123, 2524]*
***Alcohol drinkers (%)***	10.4^+++^	20.7	78.6***
***Smokers (%)***	4.17	3.45	10.7

Data given as mean ± SD, median [interquartile range] or percentage

*p<0.05

**p<0.005

***p<0.0005 vs African subjects

^**++**^p<0.005

^**+++**^p<0.0005 vs Caucasian subjects

The prevalence of the metabolic syndrome was highest in the Indian subjects, being significantly (p<0.005) higher than that observed in the Caucasian cohort. Total energy expenditure was significantly higher in the African compared to the Indian (p<0.005) and Caucasian (p<0.05) subjects, whilst the number of steps walked per day was statistically greater in African than Indian subjects (p<0.05).

### Comparison of cardiometabolic variables across hepatic fat tertiles

A comparison of blood pressure, serum lipids, glucose, insulin and insulin resistance (HOMA) across tertiles of hepatic fat content are shown in Table 2. There were no significant differences in blood pressure, lipid profile and blood glucose levels across the hepatic fat tertiles, however both insulin and HOMA levels were significantly (p<0.005 for both) higher in tertile 1 (highest hepatic fat content) when compared to tertile 3.

**Table 2:** Comparison of blood pressure, lipid, glucose, insulin and insulin resistance levels across tertiles of hepatic liver fat content.

Variables	Tertiles of hepatic fat content		
	Tertile 3 (low hepatic fat)	Tertile 2	Tertile 1 (high hepatic fat)
***Systolic bp (mmHg)***	110 [106, 120]	120 [112, 128]	118 [106, 130]
***Diastolic bp (mmHg)***	74.0 [70.0, 80.0]	80.0 [70.0, 81.0]	76.0 [70.0, 84.0]
***Triglycerides (mmol/L)***	0.78 [0.60, 1.12]	1.10 [0.74, 1.41]	1.01 [0.83, 1.49]
***Total cholesterol (mmol/L)***	4.70 [3.73, 5.25]	4.64 [4.12, 5.34]	4.75 [4.28, 5.67]
***LDL (mmol/L)***	2.56 [2.00, 3.25]	2.60 [2.02, 3.10]	2.84 [2.50, 3.41]
***HDL (mmol/L)***	1.50 [1.21, 1.90]	1.56 [1.30, 1.80]	1.44 [1.24, 1.67]
***Glucose (mmol/L)***	4.80 [4.60, 5.10]	4.76 [4.50, 5.00]	5.00 [4.50, 5.39]
***Insulin (pmol/L)***	29.1 [20.8, 52.7]	47.6 [33.2, 71.4]	67.6 [36.8, 107]**
***HOMA***	0.90 [0.63, 1.56]	1.63 [0.98, 2.22]	2.22 [1.10, 3.04]**

Data given as median [interquartile range]

**p<0.005 vs tertile 3

### Determinants of hepatic fat content and insulin resistance

Table 3 shows the final multivariable linear regression models for LAR and HOMA following backward, stepwise regression analysis. African ethnicity and subcutaneous fat were found to be significantly associated with lower levels of hepatic fat content whilst HOMA and dysglycaemia were positively associated with liver fat (see Table 3). Waist circumference was weakly (p = 0.05) and positively associated with liver fat.

**Table 3:** Multivariable linear regression models for liver: Spleen attenuation ratio and insulin resistance (HOMA).

Dependent variable	Independent variables with unstandardised β-value (p-value)	R^2^ value for model (p-value)
Liver-spleen attenuation ratio*	• African: 3.04 (0.004) • Dysglycaemia: -3.58 (0.01) • HOMA: -0.97 (0.01) • Subcutaneous fat: 0.007 (0.03) • Waist: -0.10 (0.05)	0.29 (<0.0001)
***HOMA***	• African: -0.24 (0.0009) • Visceral fat: 0.002 (<0.0001)	0.22 (<0.0001)

*The higher the liver-spleen attenuation ratio, the lower the level of hepatic fat

As shown in Table 3, the only variables that were found to be significant correlates of HOMA were African ethnicity (negative effector) and visceral fat (positive effector).

### Metabolic syndrome and intrahepatic fat

In subjects with the metabolic syndrome the levels of intrahepatic fat (LAR: 1.19 [0.89, 1.33]) were higher than in those without the syndrome (1.28 [1.17. 1.38]; p = 0.003) but, as shown in Table 3, metabolic syndrome was not a significant determinant of liver fat levels. However, the regression models shown in Table 4 demonstrate that in a univariate regression model metabolic syndrome correlates strongly with liver fat levels (model 1). This suggests that other variables must explain the relationship between metabolic syndrome and liver fat and these variables are likely to be those shown in the final linear regression model for hepatic fat (see Table 3). The data in Table 4 demonstrates that both dysglycaemia (model 2) and HOMA (model 3) individually attenuated the relationship between metabolic syndrome and liver fat levels, whilst together (model 4) these 2 variables have an even greater effect on weakening the relationship between metabolic syndrome and liver fat content. However, African ethnicity, subcutaneous fat or waist circumference if individually added to model 1, did not attenuate the correlation between metabolic syndrome and liver fat (data not shown).

**Table 4:** Multiple linear regression analysis for the identification of factors that attenuate the relationship between hepatic fat content and metabolic syndrome.

Model number	Dependent variable	Independent variables with unstandardised β-value (p-value)	R^2^ value for model (p-value)
***1***	Liver-spleen attenuation ratio*	• Metabolic syndrome: -3.72 (0.003)	0.07 (0.003)
***2***	Liver-spleen attenuation ratio	• Metabolic syndrome: -1.48 (0.33) • Dysglycaemia: -4.31 (0.01)	0.12 (0.0007)
***3***	Liver-spleen attenuation ratio	• Metabolic syndrome: -2.02 (0.14) • HOMA: -1.29 (0.002)	0.14 (0.0002)
***4***	Liver-spleen attenuation ratio	• Metabolic syndrome: -0.21 (0.89) • HOMA: -1.22 (0.003) • Dysglycaemia: -4.10 (0.02)	0.18 (<0.0001)

*The higher the liver-spleen attenuation ratio, the lower the level of hepatic fat

## Discussion

Data from the current study shows that African females, although having the greatest level of body fat, have the lowest level of hepatic fat content. Furthermore, liver fat levels are positively influenced by dysglycaemia and insulin resistance but attenuated by subcutaneous abdominal fat. Metabolic syndrome is associated with high hepatic fat content which may be due to the high levels of insulin resistance and dysglycaemia that characterize this disorder.

The high BMI among the African subjects in the present study is consistent with previous studies that showed a high prevalence of obesity in black African females [11–12]. In keeping with these findings, the African subjects also had more subcutaneous abdominal fat and a greater waist circumference than the Indian and Caucasian subjects.

Petersen et al. [20] investigated the prevalence of various metabolic risk factors amongst different racial groups and found that in spite of having lower BMI, Asian-Indian subjects had the highest levels of fasting serum insulin and insulin resistance as measured by the HOMA index in comparison to a number of other ethnic groups including African Americans. Consistent with their findings, in spite of having the lowest BMI, the Indian subjects in the current study had significantly higher insulin levels and were more insulin resistant compared to the black African subjects. Furthermore, using the harmonised guidelines [19], a higher percentage of Indian subjects fulfilled the metabolic syndrome criteria than African and Caucasian subjects.

Although liver biopsy remains the gold standard for diagnosing NAFLD, its invasiveness and cost precludes its use as a screening tool in general populations. However, imaging techniques such as ultrasound and computerized tomography (CT) have been accepted as tools for screening for fatty liver disease, and new modalities including magnetic resonance imaging (MRI) proton density fat fraction (PDFF) [21,22], transient elastography and magnetic resonance elastography (MRE) have further advanced the accuracy and usefulness of these imaging methodologies for the measurement of hepatic steatosis and fibrosis [22]. Serum levels of liver enzymes can also be used as a marker of hepatic fat deposition, however studies have shown that this method is not very sensitive. Thus, serum alanine aminotransferase (ALT) levels have been shown to be normal in 79% of patients with hepatic steatosis [23] and the complete histological range of NAFLD can be observed in patients with normal serum ALT levels [24,25]. Imaging techniques may be a more reliable method of measuring hepatic fat content with CT demonstrating a strong correlation between the liver-to-spleen attenuation ratio and the severity of steatosis [26,27]. Therefore, we chose to use the CT methodology for assessing hepatic fat content. This technique also allowed us to quantify visceral fat, which is known to be positively associated with liver steatosis [5,6].

Several studies have shown that there may be ethnic differences in the prevalence of NAFLD. Studies in the USA have shown that Hispanics had the highest and African-Americans the lowest prevalence of NAFLD [28–30]. Younossi et al., [28] reported that NASH was independently associated with being Hispanic and inversely associated with being African American, and these findings support the results of the present study where black African subjects had the lowest level of hepatic fat. This is an interesting observation given that the African subjects had the highest BMI when compared to the other 2 ethnic groups. It is known that steatohepatitis is more prominent in obese subjects [31] and our findings therefore suggest that there is some level of protection from hepatic fat deposition in obese African females. The present study is the first to compare levels of hepatic fat in an indigenous African population to that in other ethnic groups. A previous study performed in South Africa did assess hepatosteatosis via liver biopsies in a number of different ethnic groups but this was a descriptive study and only a small number of black African subjects were included [32].

The results of this study show that insulin and HOMA levels rose with increasing liver fat, and that HOMA correlated with hepatic fat in a multivariable regression model thus indicating a strong relationship between insulin resistance and hepatic steatosis, as reported in other studies [33]. Dysglycaemia and insulin resistance independently correlated with liver fat content in the linear multivariable regression model. We also observed that in a multivariable regression model for insulin resistance, hepatic fat did not appear as a significant determinant whereas visceral fat was found to be a positive influence and African ethnicity was found to be a negative influence on HOMA levels. The interaction between hepatic steatosis, insulin resistance and type 2 diabetes is a complex one with the literature suggesting a number of different possible relationships between these metabolic states including a bidirectional relationship between insulin resistance and hepatosteatosis [34], and even a dissociation of insulin resistance from hepatosteatosis [35]. However, data from the current study suggests that dysglycaemia and insulin resistance independently of each other are associated with hepatic fat content, which is not a determinant of insulin resistance. Furthermore, metabolic syndrome is associated with higher levels of liver fat content and this is due to the dual effects of insulin resistance and dysglycaemia, both of which have been proposed to enhance hepatic lipogenesis [36]. An alternative hypothesis is that other factors mediate the link between insulin resistance and dysglycaemia with hepatosteatosis, but have not been quantified in our study. One such factor is the adipokine adiponectin, which is known to reduce hepatic lipid content [37] and to be present at lower levels in subjects with type 2 diabetes and predict progression to diabetes [38].

An interesting finding of our study was that subcutaneous abdominal fat was negatively associated with the level of hepatic fat. It has been suggested that subcutaneous adipose tissue acts as a reservoir for excess triglycerides thus reducing their deposition at ectopic sites like the liver or skeletal muscle [39], and this hypothesis may explain our data. Other studies have also noted favourable effects of subcutaneous adipose tissue on metabolic function [40,41].

The results from the linear multivariable regression analysis demonstrated that physical activity, education level and smoking did not influence hepatic fat deposition. Given that African ethnicity is associated with lower levels of steatosis, independent of the influence of insulin resistance, dysglycaemia and body fat distribution, these results suggest that ethnic differences in liver fat levels are not explained by any of the behavioural, socio-demographic or biological factors measured in this study and must therefore be related to other uncaptured variables. Also, the alcohol intake recorded in this study was described as ‘occasional’ by all participants who did consume alcohol, and was not associated with hepatic fat content in the regression model. Thus, the liver fat deposition recorded in our study was likely not due to alcoholic steatohepatitis.

A number of studies have demonstrated that higher levels of physical activity can improve steatosis. Thus, exercise intervention studies and cross sectional studies in which physical activity was assessed have all shown that various markers of steatosis are improved [42]. However, in the present study no effect of physical activity on hepatic fat content was observed. This may be related to the small sample size of our study and also to differences in methodologies between the present and previous studies. Thus, many of these investigations used serum liver enzyme levels as markers of steatosis rather than direct measures of hepatic fat content or quantified physical activity using questionnaires rather than by the use of activity monitors. However, the activity monitors were worn for only 7 days and therefore the data obtained from these instruments may not be representative of physical activity over extended periods of time. Therefore, the lack of association between hepatic fat content and physical activity that was observed in the current study must be viewed in the context of the limitations of the method used for the measurement of physical activity.

The genetic aetiology of NAFLD has been studied in a number of different populations groups. A recent meta-analysis of such studies, but focussing on the relationship of the rs738409 polymorphism of the patatin-like phospholipase domain-containing 3 (PNPLA3) gene, showed a strong relationship of this polymorphism with NAFLD [43]. This association was observed across a number of different ethnic groups. However, no studies on the association of variants in the PNPLA3 gene with NAFLD have been conducted in African populations and no data is currently available on the genetic determinants of liver fat content in any indigenous sub-Saharan African populations. Gene association analyses were not conducted in the current investigation, but are an obvious target of future investigations.

The current study was cross-sectional in format and used regression models to delineate the modulators of hepatic fat levels. Thus, we were not able to isolate the true determinants of liver fat content using this protocol. However, the regression models did uncover some interesting associations which should be investigated in greater detail in future studies. Further drawbacks of this study was that we did not measure serum adipokine concentrations, in particular adiponectin and alcohol consumption was assessed using a simple questionnaire rather than measuring blood markers of alcohol intake. Additionally, the sample size for this study was relatively low but was still sufficiently powered to detect significant and important associations, and the study participants were recruited by convenience sampling and therefore may not be representative of the total population. The strength of this study was that it was the first to measure hepatic fat content in an indigenous African population relative to other ethnic groups and included the measurement of a broad range of biological, behavioural and demographic variables appropriate to the aims of the study.

In summary, we have shown that the level of hepatic lipid deposition is lower in African compared to Asian Indian and Caucasian women due to unknown factors despite the higher BMI of the African population. Dysglycaemia and insulin resistance are both independently associated with greater levels of liver fat whereas subcutaneous abdominal adipose tissue is negatively associated with hepatic fat content. Insulin resistance is not influenced by the level of steatosis. These observations must be further investigated in longitudinal surveys.

## Supporting information

S1 Data Data file Naran et al. Data file containing all study data.
(XLSX)
